# The SURgical PAtient Safety System (SURPASS) checklist optimizes timing of antibiotic prophylaxis

**DOI:** 10.1186/1754-9493-4-6

**Published:** 2010-04-13

**Authors:** Eefje N de Vries, Lucia Dijkstra, Susanne M Smorenburg, R Peter Meijer, Marja A Boermeester

**Affiliations:** 1 Department of Surgery, Academic Medical Centre, Amsterdam, the Netherlands; 2 Department of Quality and Process Innovation, Academic Medical Centre, Amsterdam, the Netherlands; 3 Department of Anaesthesiology, Academic Medical Centre, Amsterdam, the Netherlands

## Abstract

**Background:**

Surgical site infection (SSI) is an adverse event in which a close relation between process of care and outcome has been demonstrated: administration of antibiotic prophylaxis decreases the risk of SSI. In our tertiary referral centre, a SURgical PAtient Safety System (SURPASS) checklist was developed and implemented. This multidisciplinary checklist covers the entire surgical pathway and includes, among other items, administration of antibiotic prophylaxis before induction of anaesthesia. The aim of this study was to determine the effect of SURPASS implementation on timing of antibiotic prophylaxis.

**Methods:**

A retrospective analysis was performed on two cohorts of patients: one cohort of surgical patients that underwent surgery before implementation of the checklist and a comparable cohort after implementation. The interval between administration of antibiotic prophylaxis and incision was compared between the two cohorts.

**Results:**

A total of 772 surgical procedures were included. More than half of procedures were gastro-intestinal; others were vascular, trauma and hernia repair procedures. After implementation, the checklist was used in 81.4% of procedures. The interval between administration of antibiotic prophylaxis and incision increased from 23.9 minutes before implementation of SURPASS to 29.9 minutes after implementation (p = 0.047). In procedures where the checklist was used, the interval increased to 32.9 minutes (p = 0.004). The proportion of patients that did not receive antibiotics until after the incision decreased significantly.

**Conclusion:**

The use of the SURPASS checklist leads to better compliance with regard to the timing of antibiotic prophylaxis administration.

## Introduction

Surgical site infection (SSI) is a common complication of surgery: reported incidence rates range from 2% to 20%, the variation largely depending on case mix [1-3]. SSIs are a major cause of morbidity, mortality and healthcare costs [4-6]. While in many adverse events, the connection between process of care and outcome is hard to define, this is not the case with SSI. Among many process measures that decrease SSI rates, the effect of preoperative administration of antibiotic prophylaxis (AP) has been demonstrated most extensively [7-10]. 

Much has been written about the optimal time frame of AP. Studies suggest that AP administration should be as close to the incision as possible, while agreeing that some time must be allowed for adequate tissue concentration to be built up [11-13]. In 1992, Classen et al showed that the greatest risk reduction for SSI occurred when antibiotics were administered within two hours before the incision, as opposed to administration after incision or more than two hours prior to the incision [14]. Since then, a number of studies have been published that attempted to further define this interval [15-18]. A recent publication by Weber et al included 3,836 general surgical procedures; the greatest risk reduction occurred when antibiotics were administered between 30 and 60 minutes prior to the incision [18]. In contrast, a study by Steinberg et al, including 4,472 cardiac, orthopaedic and gynaecological patients, showed that the risk of SSI was lowest when antibiotics were administered within the final 30 minutes pre-incision, a difference that did not reach statistical significance [16].

Most guidelines recommend administration within one to two hours of incision [19-21]. However, the practical implementation of these recommendations has been shown to be difficult [22-27]. In some settings, antibiotics have been administered too early [28-30]; in most, too late or not at all [22,23,25].

To increase standardization in the surgical pathway and improve surgical patient safety, a SURgical PAtient Safety System (SURPASS) checklist was developed using all relevant literature and subsequently validated by observation of the surgical pathway and practical evaluation [31]. This multidisciplinary checklist covers the entire surgical pathway and includes, among many other items, administration of AP in the operating room before induction of anaesthesia. The aim of this study was to determine the effect of SURPASS implementation on timing of AP.

## Methods

### Setting

This study was performed in a tertiary referral centre. In this centre, AP is given in the operating room (OR) and the standard for the timing of AP is ≥30 minutes before the incision. Most commonly, the anaesthesiologist will insert an intravenous line once the patient has arrived in the operating room. Before the implementation of the checklist, in some cases, antibiotics would be administered by the anaesthesiologist at the start of induction. More often, antibiotics would not be administered until the start of surgery, when the surgeon ordered AP to be given.

### Implementation

Over the course of a year (Jan-Dec 2008), the entire SURPASS checklist was implemented in a step-by-step fashion in the surgical wards and operating rooms. From January 2008 onwards, the time out part of SURPASS was implemented in the OR. This part consists of a short discussion to be performed in the operating room before induction of anaesthesia. One of the 16 items to be checked by the surgeon, anaesthesiologist and operating assistant during this discussion is: 'Appropriate antibiotic prophylaxis administered ≥30 minutes before incision'. All completed checklists were prospectively registered in the electronic registration system in use in the operating rooms.

### Study design

A retrospectively defined cohort of patients that underwent surgery prior to the implementation of the checklist was compared to a cohort that underwent surgery after implementation. For two three-month periods (October-December 2007 vs. October-December 2008), all surgical procedures where AP was indicated by protocol were analysed. Both elective and urgent procedures were included. In the post-intervention cohort, all procedures were included, whether or not the checklist had been used. This strategy was chosen in order to provide a realistic estimate of the potential effect of the intervention, taking into account a less than 100% compliance rate.

### Data collection

Registration of data was partly prospective. For all procedures, the time of incision was extracted from the electronic registration system in use in the operating rooms. In 2008, an electronic patient data management system was implemented for the anaesthesiologists in the OR and recovery room. For the post-intervention cohort, data on timing and choice of antibiotics were extracted from these electronic anaesthesia records. For the pre-intervention cohort, data on timing and choice of antibiotics were extracted from the paper anaesthesia records. Reliability of retrospectively collected data was checked by observation of a new sample of procedures, in which actual times of antibiotic administration and incision were compared to electronically registered times.

### Analysis

Intervals between administration of AP and incision were calculated. The means of the two cohorts were compared using the independent samples T test. In addition, the proportion of patients that received AP after the incision was assessed and the difference between the two cohorts was calculated using the Chi-Square test.

In a second analysis, all procedures where the checklist had not been used were excluded from the post-intervention cohort, to identify the actual effect of the checklist in case of a 100% compliance rate. All statistical analyses were two-tailed and values of p < 0.05 were considered significant. The analyses were completed using Statistical Package for the Social Sciences version 15.0 (SPSS^®^, Chicago, Illinois, USA).

## Results

**Figure 1:**
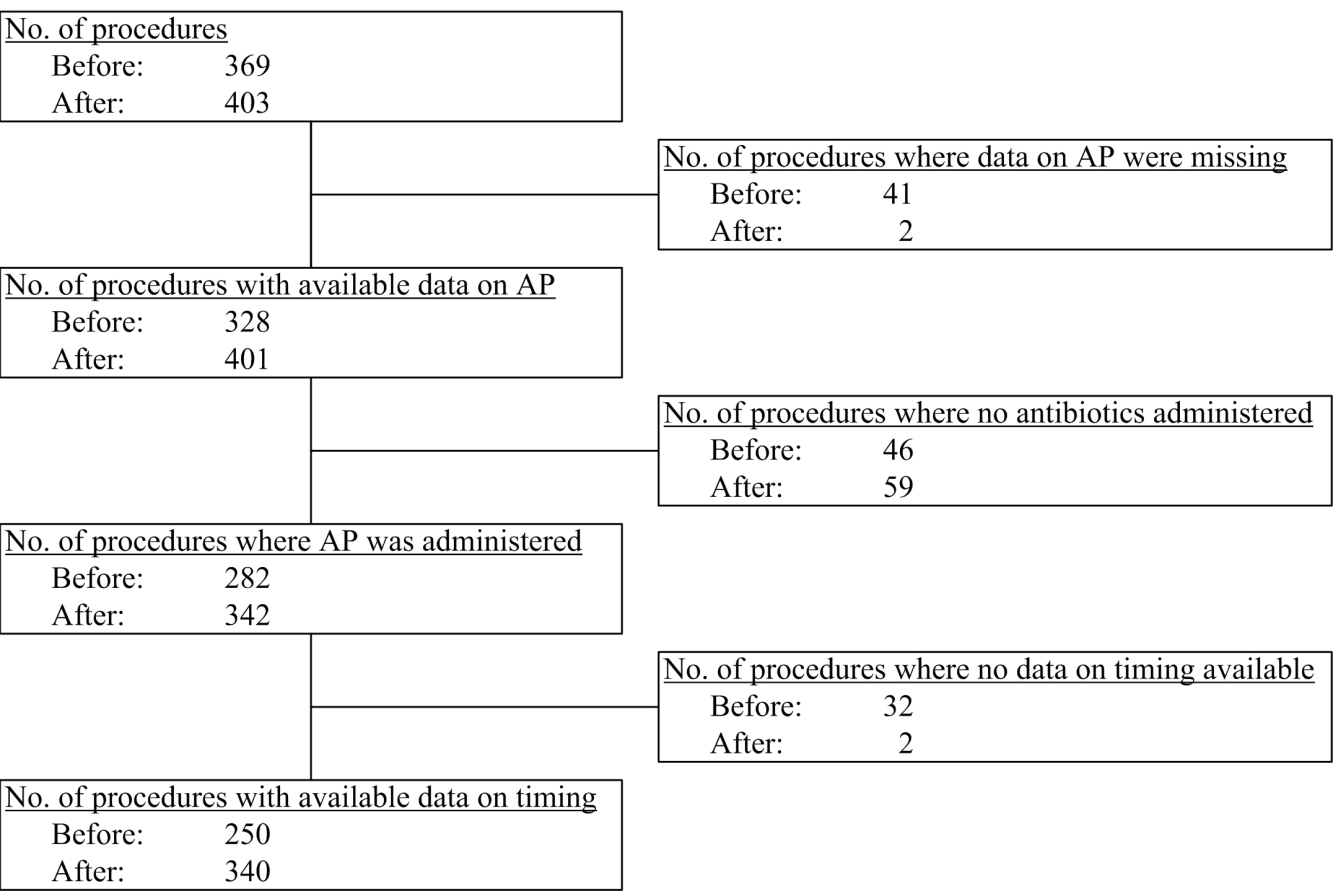
**Flowchart of inclusion before and after checklist implementation**.

A total of 772 surgical procedures were included (figure 1). In the post-implementation cohort, the checklist was actually used in 328 of 403 procedures (81.4%). Patient characteristics are listed in table 1. More than half of procedures were gastro-intestinal; others being vascular, trauma and hernia repair procedures. Data on antibiotic prophylaxis were available in 729 procedures. The percentage of patients that did not receive antibiotics did not differ between the two groups (table 1). Most patients (45.7%) received cefuroxime and clindamycin; a third of patients received a single dose of cefuroxime.

**Table 1:** Patient characteristics and antibiotic prophylaxis.

Patient characteristics		Pre-intervention (N = 369)	Post-intervention (N = 403)	p
Male		59.3%	55.3%	0.260
Age		55.3 ± 18.0	53.6 ± 18.3	0.191
Urgent (surgery required within one day)		33.1%	27.0%	0.068
Category of operation	Gastrointestinal	52.0%	51.1%	0.286
	Trauma	22.5%	25.3%	
	Vascular	20.9%	16.9%	
	Hernia repair	4.6%	6.7%	

**Antibiotic prophylaxis**		**Pre-intervention** **(N = 328)**	**Post-intervention** **(N = 401)**	

No antibiotics*		10.4%	11.2%	0.711
Antibiotic of choice	Cefuroxime/clindamycin	48.2%	43.6%	0.051
	Cefuroxime	30.1%	30.7%	
	Cefamandol	17.4%	15.8%	
	Other	4.3%	9.9%	

* Patients that had received therapeutic antibiotics within 24 hours preoperatively because of a pre-existing infection were excluded from this category (12 vs 14 patients)

To validate the time points that were collected from the electronic databases, a sample of 44 procedures was observed. The median difference between registered and observed time of antibiotic administration was -1.0 minute (interquartile range (IQR) -6.6 to 4.0), and the median difference between registered and observed time of incision was -0.5 minutes (IQR -2.0 to 1.0).

**Figure 2:**
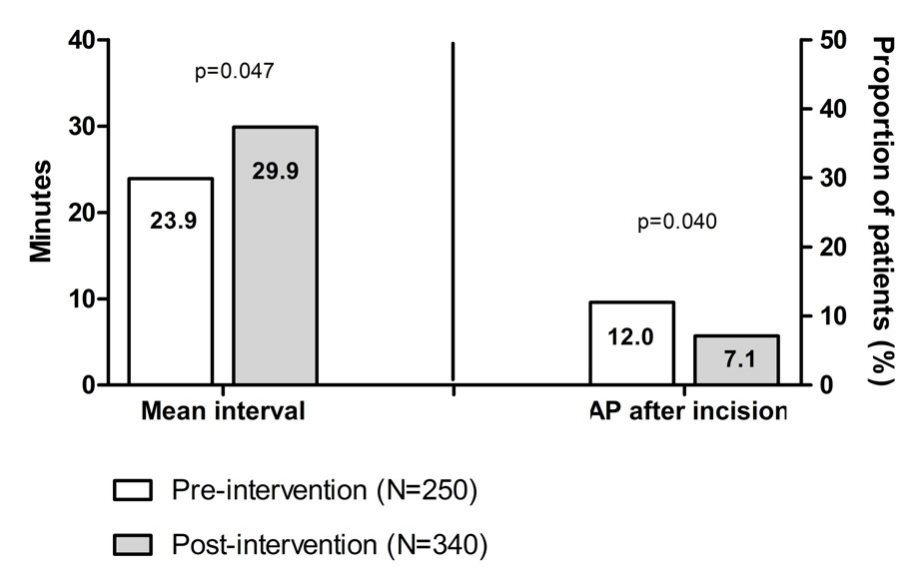
**Interval between antibiotics administration and incision before and after implementation of SURPASS**.

Data on timing of antibiotic prophylaxis was available in 590 procedures. The mean interval between administration of AP and incision increased from 23.9 minutes (standard deviation 37.1) before implementation of the checklist to 29.9 minutes (standard deviation 31.9) after implementation (p = 0.047) (figure 2). The proportion of patients that did not receive antibiotics until after the incision decreased from 12.1% to 7.1% (p = 0.04).

**Figure 3:**
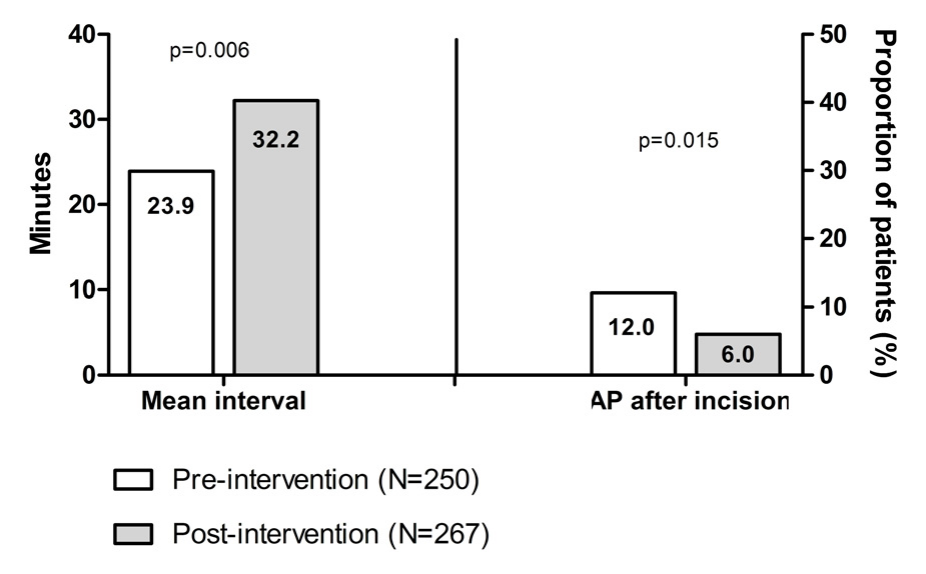
**Interval between antibiotics administration and incision when looking only at procedures where the checklist was actually used**.

When looking only at procedures where the checklist was actually used, the post-implementation interval increased to 32.2 minutes (p = 0.006; figure 3). In this cohort, the proportion of patients that did not receive antibiotics until after the incision decreased to 6.0% (p = 0.015).

## Discussion

Although it has been shown repeatedly that timely administration of AP decreases the incidence of SSI, the implementation of this knowledge into daily practice remains problematic. This study showed that implementation of a comprehensive surgical safety checklist (SURPASS) significantly improved compliance with hospital standards for timing of AP administration. The proportion of patients that did not receive antibiotics until after the incision decreased significantly.

Remarkably, the proportion of patients that did not receive AP, thus deviating from protocol, did not change. This might reflect deliberate divergence from protocol, as there were no differences in case mix before and after implementation. The inclusion criteria were based on groups of surgical procedures, without taking into account individual patient characteristics. In the 10% of patients that did not receive antibiotic prophylaxis, this might well have been a deliberate and well-considered choice, for example in the case of correction of a ventral hernia, where antibiotic prophylaxis is not always mandatory.

The checklist was not always used correctly: since the item on the checklist reads: 'Appropriate antibiotic prophylaxis administered ≥30 minutes before incision', one would expect AP to have been administered at least 30 minutes before incision in all procedures where the checklist had been used. This was obviously not the case. Apparently, the item was sometimes completed before AP had been administered and the 30 minute interval could not be guaranteed afterwards. This suboptimal way of checklist use might be solved with more education.

The optimal AP-incision interval to prevent SSIs has not been firmly established yet. Over 30 years ago, it was shown that administration within 1 hour prior to the incision yielded better results than administration 8 to 12 hours before incision [32]. In 1992, Classen et al showed that administration within two hours to incision is preferable to administration before or after this period [14]. Two recent studies attempting to further define this interval presented conflicting results [16,18]. However, all available studies agree that AP administration after incision is undesirable; the SURPASS significantly decreased the proportion of patients receiving AP after incision. In addition, the mean AP-incision interval increased to within hospital standards.

Other studies have described interventions to improve timing of AP administration, usually in the context of multiple simultaneous interventions to decrease SSI rates. In a multicentre improvement project, a number of interventions were performed, consisting mostly of educational activities and generation of feedback data [24]. In that study, the proportion of patients receiving antibiotics within one hour preoperatively improved by 15%. Another quality improvement project focused mainly on changes in workflow management and the assignment of responsibilities [27]. Compliance with national guidelines improved by 20%. In an orthopaedic setting, timely administration of AP was added to a time out protocol to prevent wrong site surgery, leading to a 34% increase in the proportion of patients that received antibiotics within one hour preoperatively [25]. The intervention last mentioned resembles the present study, although one crucial difference is that the time out procedure described by Rosenberg et al takes place just before the incision, whereas the time out procedure included in the SURPASS checklist must be performed before the start of anaesthesia. This by itself can lead to an interval of at least 30 minutes in most procedures (assuming the checklist is used correctly), in particular in centres with more complex surgery, where anaesthetic preparation can take a considerable amount of time.

Although SURPASS is the only validated comprehensive checklist for the entire surgical patient pathway, another surgical checklist consisting of an extended time out procedure was recently described by a working party of the World Health Organization [33]. One of the items on that checklist was 'administration of AP within 60 minutes of incision', but this item was not checked until directly before incision. The authors report that mean rates of AP administration rose from as low as 56.1% (with a lower range of 18.1%) to 82.6%, and that SSI rates dropped from 6.2% to 3.4%. However, this study included a number of low-income countries with high baseline rates of SSI, where the effect of using proper AP to start with would have had a large impact. Considering the baseline SSI rate of approximately 5% and the baseline AP compliance rate of 90% in our centre, the number of patients included in the present study was too small to be able to detect a relevant influence of the checklist on SSI rates.

This study has several limitations. First, it is a before-after study: any change that was observed might have been influenced by secular changes, for example, the introduction of electronic anaesthesia records. However, we have no reason to suspect the introduction of electronic anaesthesia records had any influence on timing or recording of antibiotic prophylaxis, as it was merely a registration system and did not provide any prompts. In addition, a randomized study was not feasible because in interventions that aim to change human behaviour, there will always be contamination between intervention and control arm: whether consciously or not, the care that is provided to non-checklist patients will be influenced by the checklist. A parallel design comparing different hospitals would have been hampered by uncertainties related to inter-hospital and case mix differences. Second, the difference in data collection regarding timing of antibiotics before and after the intervention might have been of influence. In the pre-intervention cohort (paper records), data on timing of AP were missing in 73 patients, whereas, in the post-intervention cohort (electronic records), data were available in all but 4 patients. However, a difference in timing between procedures where data on timing were available and procedures where these data were not available, was deemed unlikely. Third, the fact that data on timing were collected retrospectively might render them less reliable; however, method validation in a separate small cohort comparing electronic recording with simultaneous observation showed negligible differences. Fourth, and most importantly, the effect of the SURPASS checklist on patient outcomes such as SSI's was not assessed in this study, because the numbers were too small to be able to detect a decrease in SSI's. Whether the implementation of SURPASS will lead to a decrease in SSI's will have to be tested in a much larger cohort of patients.

## Conclusions

In this study, implementation of the multidisciplinary SURPASS checklist improved compliance with hospital standards for the timing of antibiotic prophylaxis. The proportion of patients that did not receive antibiotics until after the incision decreased significantly.

The checklist is currently being adopted in numerous hospitals, both in the Netherlands and in other European countries.

## Competing interests

The authors declare that they have no competing interests.

## Authors' contributions

EdV conceived of the study, analysed the data and wrote the first draft of the manuscript. LD collected the data, assisted in analysing the data and assisted in writing the first draft of the manuscript. SM participated in the design of the study. RM participated in the design of the study and facilitated data collection. MB conceived of the study, participated in the design and analysis and helped draft the manuscript. All authors read and approved the final manuscript.
